# Efficient generation of a single-copy eft-3p::TIR1::F2A:: BFP::AID*::NLS allele in the *C. elegans ttTi5605* insertion site** through recombination-mediated cassette exchange

**DOI:** 10.17912/micropub.biology.000425

**Published:** 2021-08-03

**Authors:** An A. Vo, Max T. Levenson, James Matthew Ragle, Jordan D. Ward

**Affiliations:** 1 Department of Molecular, Cell, and Developmental Biology, University of California – Santa Cruz, Santa Cruz, CA, USA

## Abstract

The auxin-inducible degron (AID) system is a widely used system to conditionally deplete proteins. Using CRISPR/Cas9-based genome editing in *C. elegans*, we recently generated a set of single-copy, tissue-specific and pan-somatic TIR1-expressing strains carrying a BFP reporter inserted in single-copy into two commonly used, well-characterized genetic loci. However, we were unable to obtain a strain carrying a pan-somatic eft-3p::TIR1::F2A::BFP::AID*::NLS transgene inserted into the chromosome II *ttTi5605 *insertion site. Using recombination-mediated cassette exchange (RMCE) we were able to efficiently obtain this knock-in. The resulting strain displayed equivalent depletion of an AID*::GFP reporter compared to our previously generated eft-3p::TIR1::F2A::BFP::AID*::NLS transgene knocked into the chromosome I *ttTi4348 *insertion site. This work highlights the power of RMCE for generating new reagents for the AID system and provides an eft-3p::TIR1::F2A::BFP::AID*::NLS allele on chromosome II which will simplify genetic crossing schemes when using the AID system.

**Figure 1. Validation of a chromosome II single-copy eft-3p::TIR1::F2A::BFP::AID*::NLS transgene:**
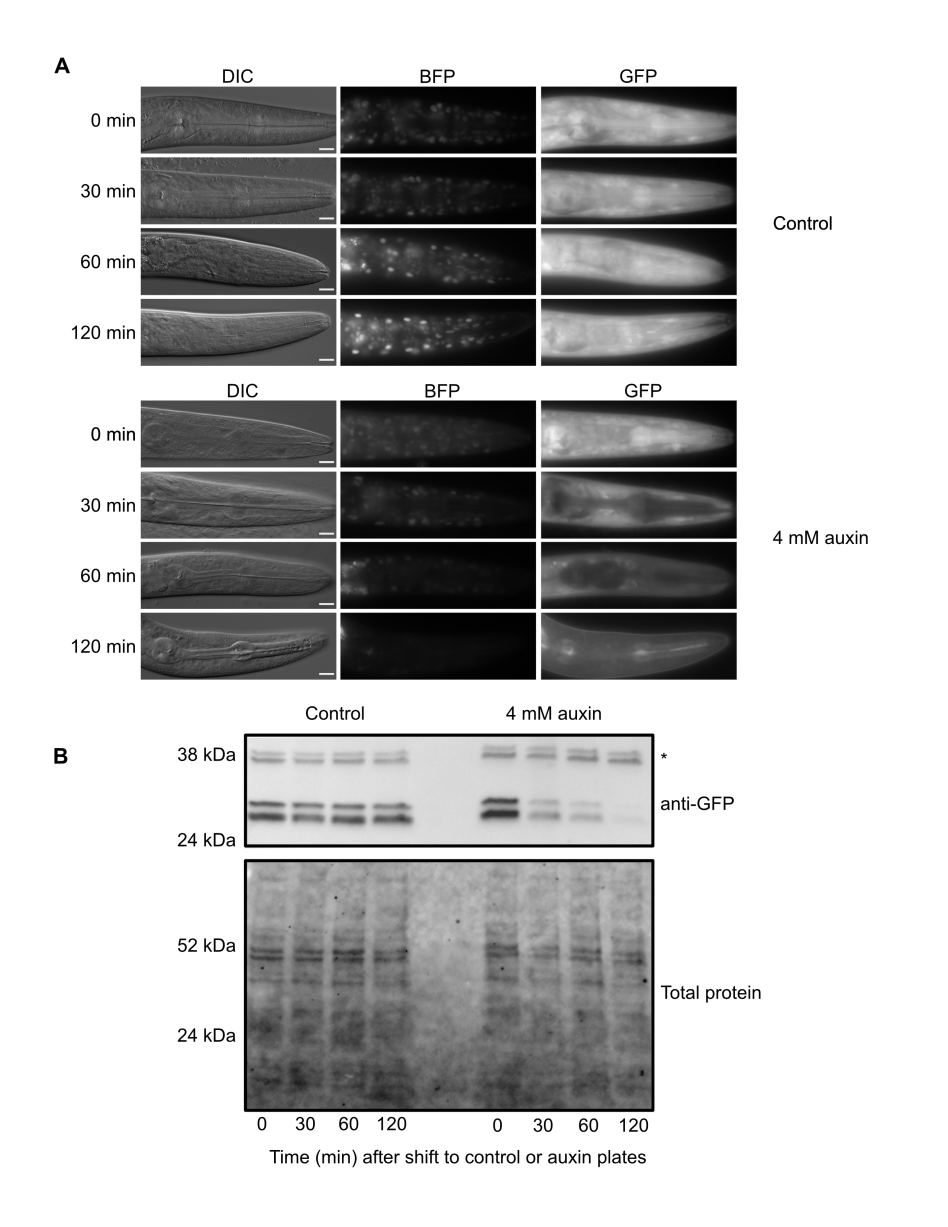
eft-3p::TIR1::F2A::BFP::AID*::NLS animals (late L3 stage) were shifted onto control or 4 mM K-NAA (1-Naphthaleneacetic Acid, Potassium Salt) auxin plates for the indicated times (0, 30, 60, 120 minutes). (A) Animals were collected and DIC, BFP, and GFP imaging of the head region was performed. Scale bars represent 10 µm. (B) Western blots detecting AID*::GFP in control- or auxin (4 mM K-NAA)-treated animals. Anti-GFP blots (top) detected background bands (marked with *) also reported by Ashley **et al.*,* 2021 and a doublet at approximately 27 kDa, consistent with the predicted size of GFP. Total protein is provided as a loading control. Equal exposure times were used to collect each set of GFP and BFP images.

## Description

The AID system has emerged as a powerful tool to conditionally deplete proteins in a wide-range of organisms and cell types (Nishimura *et al.* 2009; Holland *et al.* 2012; Zhang *et al.* 2015; Natsume *et al.* 2016; Trost *et al.* 2016; Brown *et al.* 2017; Daniel *et al.* 2018; Chen *et al.* 2018; Camlin and Evans 2019). The system is comprised of two components. A plant F-box protein Transport Inhibitor Response 1 (TIR1) is expressed and forms a complex with endogenous Skp1 and Cul1 proteins to form a functional SCF ubiquitin ligase (Nishimura *et al.* 2009; Natsume and Kanemaki 2017). TIR1 can either be expressed constitutively or in a tissue-specific manner depending on promoter choice. A degron sequence from the IAA17 protein is fused to the protein of interest (Nishimura *et al.* 2009; Natsume and Kanemaki 2017). Commonly used auxin-inducible degrons include 44 amino acid (AID*) and 68 amino acid (mAID) fragments of IAA17 (Morawska and Ulrich 2013; Li *et al.* 2019). Addition of the plant hormone auxin bridges an interaction between TIR1 and the degron and the SCF ligase ubiquitylates the degron-fused protein leading to proteasomal degradation.

We recently described a new set of strains and reagents for the *C. elegans* AID system (Ashley *et al.* 2021), complementing the original version of the system, which used an mRuby2 fusion to monitor the expression of TIR1 (Zhang *et al.* 2015). We developed a TIR1::F2A::BFP::AID*::NLS transgene (Ashley *et al.* 2021) in which an F2A ribosome skip sequence allows production of separate TIR1 and BFP::AID*::NLS proteins from a single mRNA transcript (Ryan and Drew 1994; Ryan *et al.* 1999; Donnelly *et al.* 2001; de Felipe *et al.* 2003; Ahier and Jarriault 2014). The use of a BFP reporter allows for depletion experiments while imaging red and green fluorescent proteins (Ragle *et al.* 2020). TIR1 expression can be monitored by nuclear-localized BFP and the AID* sequence allows detection of TIR1 activity by auxin-dependent BFP depletion (Ashley *et al.* 2021). We previously inserted tissue-specific (germline, intestine, neuron, muscle, hypodermis, seam cell) TIR1::F2A::BFP::AID*::NLS transgenes into standardized genetic loci on chromosomes I and II (ttTi4348 and ttTi5605 Mos insertion sites, respectively) using CRISPR/Cas9-mediated genome editing (Frøkjaer-Jensen *et al.* 2008; Frøkjær-Jensen *et al.* 2012; Ashley *et al.* 2021). We generated a pan-somatic eft-3p:: TIR1::F2A::BFP::AID*::NLS transgene in the chromosome I site (Ashley *et al.* 2021), but were unable to generate an equivalent insertion in chromosome II despite injecting over two hundred animals. All other insertions were obtained by injecting 60 animals or fewer. As the pan-somatic TIR1 strains are widely requested (Aric Daul, *Caenorhabditis* Genetics Center (CGC), personal communication) we attempted to generate a chromosome II pan-somatic TIR1 strain through recombination-mediated cassette exchange (RMCE), an alternate genome editing method (Nonet 2020).

RMCE uses a germline-expressed Flp recombinase to insert transgenes into specialized integration landing sites (Nonet 2020). As there are now a number of landing sites, a single integration vector can be used to generate insertions into multiple genomic loci. We generated an integration vector containing an eft-3p::TIR1::F2A::BFP::AID*::NLS transgene, injected it into a strain carrying a landing pad in the ttTi5605 insertion site in chromosome II (M. Nonet, https://sites.wustl.edu/nonetlab/rmce-insertion-strains), and recovered a single knock-in from 30 injected animals on our first attempt. We expect that we can improve on this knock-in rate and move closer to the reported efficiency of injecting seven animals to achieve a knock-in (Nonet, 2020). We then generated an eft-3p::TIR1::F2A::BFP::AID*::NLS; eft-3p::AID*::GFP strain and performed a depletion time course to validate the TIR1 allele. At the 0 minute timepoint, we observed nuclear-localized BFP, indicating that the eft-3p::TIR1::F2A::BFP::AID*::NLS transgene was being transcribed and translated (Figure 1A). In control treated animals we observed no detectable change in BFP or GFP by microscopy (Figure 1A). In contrast, animals exposed to 4 mM auxin displayed a severe reduction of BFP and GFP by 120 minutes post-treatment (Figure 1A). We confirmed these results with an anti-GFP western blot (Figure 1B). GFP levels were stable in control animals, but dropped after 30 minutes of auxin exposure and were almost undetectable after two hours of exposure, similar to what we reported for our chromosome I eft-3p:: TIR1::F2A::BFP::AID*::NLS transgene (Ashley *et al.* 2021). This new TIR1 strain should complement our original chromosome I eft-3p::TIR1::F2A::BFP::AID*::NLS strain and provide a new allele on a separate chromosome to facilitate genetic crossing schemes. This work also highlights the effectiveness of the RMCE method for generation of single-copy transgenes at defined loci (Nonet 2020).

## Methods

*Worm culture*

Animals were maintained on MYOB (Church *et al.* 1995) seeded with OP50, similar to previously described methods (Brenner 1974). 4 mM auxin plates were made by diluting 20 µl of 1 M K-NAA (1-Naphthaleneacetic Acid, Potassium Salt, Phyto-Technology Laboratories, N610)(Martinez and Matus 2020; Martinez *et al.* 2020) in 100 µl of M9 and adding to a well of a 6-well MYOB plate seeded with OP50 (5 ml volume). The plate was allowed to dry for two hours before use. For the time course experiments in Figure 1, JDW346 animals were synchronized by alkaline bleaching and released on 10 cm MYOB plates at a density of 2000 worms/plate for 26 hours at 25ºC. Animals were then shifted to 6-well plates (control or 4 mM auxin). 480 animals/well were plated to collect for western blots, 72 animals/well were plated to collect for imaging. Animals were collected at 0, 30, 60, and 120 minutes following the shift onto control or auxin plates. Animals were late L3 at the start of the time course as determined by animal size and examining vulval precursor cells.

*Imaging and analysis*

Animals were picked into a 20 µl drop of M9+5 mM levamisole on a 2% agarose pad and secured through a cover slip. Images were acquired using a Plan-Apochromat 63x/1.40 Oil DIC lens on an AxioImager M2 microscope (Carl Zeiss Microscopy, LLC) equipped with a Colibri 7 LED light source and an Axiocam 506 mono camera. Acquired images were processed through Fiji software (version: 2.0.0- rc-69/1.52p).

*Western blotting*

Western blots were performed as described in Ashley **et al.*,* 2021. At each timepoint 480 animals were washed out of a well of a control or auxin plate with 1 ml of M9+0.05% gelatin. Animals were transferred to a 1.5 ml tube, washed twice in M9+0.05% gelatin, pelleted, resuspended in 40 µl of M9+0.05% gelatin, and freeze-cracked in liquid nitrogen twice. Laemmli sample buffer was added to 1X and samples were heated to 95ºC for 10 minutes. Ten microliters of lysate were resolved by SDS-PAGE using a precast 4-20% Mini-Protean TGX Stain Free Gel (Bio-Rad) before being transferred to a polyvinylidene difluoride membrane by semi-dry transfer with a TransBlot Turbo (Bio-Rad). Total protein, pre- and post-transfer, was monitored using the stain-free fluorophore as described (Posch *et al.* 2013; Ward 2015). Stain-free imaging of total protein was used to confirm equal loading. The blot was sequentially probed with rabbit anti-GFP antibody (Abcam, #ab290) diluted 1:1000 and a goat anti-Rabbit-HRP (Kindle Biosciences LLC, #R1006) diluted at 1:1000. SuperSignal™ Western Blot Substrate (Thermo Fisher Scientific, A45916) was added and the blot was imaged with a Bio-Rad ChemiDoc imaging system.

*RMCE strain*

NM5340 *jsSi1579 [loxP rpl-28p FRT GFP::his-58 FRT3] II; unc-119(ed3) III; bqSi711 [mex-5p::FLP::SL2::mNG + unc-119(+)] IV* was a gift from Dr. Michael Nonet and will be described elsewhere. For future RMCE experiments, strain NM5402 is recommended as it has the *unc-119(ed3)* allele outcrossed. The sequence of the *jsSi1579* landing pad can be found on the Nonet lab website (https://sites.wustl.edu/nonetlab/rmce-insertion-strains/ -last edited 6-16-2021) and is inserted at an sgRNA within 50 basepairs away from the ttTi5605 insertion site.

Generation of an eft-3p:TIR1:F2A::BFP:AID*::NLS::tbb-2 3’UTR transgene

eft-3p:TIR1:F2A::BFP::AID*::NLS::tbb-2 3’UTR was PCR amplified using Phusion polymerase from pJW1441 (Ashley *et al.* 2021) using oligos 5530+5545 and the resulting product was DpnI digested and cloned into pLF3FShC using SapTrap (Schwartz and Jorgensen 2016; Nonet 2020). The plasmid was miniprepped using a Purelink Quick Plasmid Miniprep kit (Invitrogen, K210011) and injected into NM5340 germlines at 25 ng/µl. Candidate insertions were recovered as previously described (Nonet 2020). We outcrossed our initial insertion strain (JDW309) two times against a wildtype N2 strain to remove the *unc-119(ed3)* allele and the *bqSi711 [mex-5p::FLP::SL2::mNG + unc-119(+)] IV* transgene and generate JDW324. We confirmed loss of the *bqSi711* transgene by a lack of mNeonGreen expression in the germline and loss of the *unc-119(ed3)* allele through wild-type animal morphology and movement. JDW324 L1 larvae were then heat-shocked to remove the SEC as previously described (Dickinson *et al.* 2015; Nonet 2020) which generated JDW313. JDW346 was made by crossing CA1204 males to JDW324. *ieSi58* and *jsSi1579 wrdSi63* were both homozygosed before the self-excising cassette was removed by heat-shock. The excised transgene can be genotyped by triplex PCR with oligos 6282+6284+6285 (see Reagents table) using a 60ºC annealing temperature. The WT allele produces a 541 bp band, the SEC-excised knock-in produces a 343 bp band, and the primers genotype heterozygotes

## Reagents

**Table:** 

**Strain**	**Genotype**	**Available from**
N2	Wild type	CGC
NM5340	*jsSi1579 [loxP rpl-28p FRT GFP::his-58 FRT3] II; unc-119(ed3) III; bqSi711 [mex-5p::FLP::SL2::mNG + unc-119(+)] IV*	Dr. Michael Nonet
CA1204	unc-119(ed3) III; ieSi58 [eft-3p::AID*::GFP::unc-54 3’UTR + Cbr-unc-119(+)] IV.	CGC
JDW309	*jsSi1579 wrdSi57 [eft-3p:TIR1:F2A:mTagBFP2::* AID*::NLS:tbb-2 3’UTR+SEC, II:0.77] II; bqSi711 [mex-5p::FLP::SL2::mNG + unc-119(+)] IV	Dr. Jordan Ward
JDW313	*jsSi1579 wrdSi58 [eft-3p:TIR1:F2A:mTagBFP2::* AID*::NLS::tbb-2 3’UTR, II:0.77]	CGC
JDW324	*jsSi1579 wrdSi57 [eft-3p:TIR1:F2A:mTagBFP2::* AID*::NLS::tbb-2 3’UTR+SEC, II:0.77]	CGC
JDW346	jsSi1579 wrdSi63[eft-3p::TIR1::F2A::mTagBFP2::AID*::NLS::tbb-2 3’UTR, II:0.77]; ieSi58 [eft-3p::AID*::GFP::unc-54 3’UTR + Cbr-unc-119(+)] IV.	Dr. Jordan Ward

**Table:** 

**Plasmid**	**Genotype**	**Description**
pLF3FShC	*loxP SapI MCS FRT3 FRT hygR sqt-1 hs cre*	Vector for RMCE integration. From Nonet, 2020. Available from AddGene (plasmid #15083)
pJW1441	*eft-3p:TIR1:F2A:mTagBFP2*	Vector for inserting *eft-3p:TIR1:F2A:mTagBFP2* transgene by CRISPR/Cas9. From Ashley **et al.*,* 2021.
pJW2154	*eft-3p:TIR1:F2A:mTagBFP2*	Vector for inserting *eft-3p:TIR1:F2A:mTagBFP2* transgene by RMCE. This study.

**Table:** 

**Oligo**	**Sequence**
5530	5′- actataggggatatcagctggatggcgctcttcgtactgagacttttttcttggcggcac -3′
5545	5′- agtcttaagctcgggccccaaataatgctcttcgtgggcacctttggtcttttattgtcaacttc -3′
6282	5′- agctccaattcgcccgtataac -3′
6284	5′- attgtttgacctggcggaac -3′
6285	5′- atgcaagacacccgggtttg -3′
